# Feasibility of Nonintubated Anesthesia for Lumboperitoneal Shunt Implantation

**DOI:** 10.3390/clinpract12030049

**Published:** 2022-06-16

**Authors:** Abel Po-Hao Huang, Feng-Fang Tsai, Chien-Chia Chen, Tzong-Shiun Lee, Lu-Ting Kuo

**Affiliations:** 1 Division of Neurosurgery, Department of Surgery, National Taiwan University Hospital, Taipei 100, Taiwan; how.how0622@gmail.com; 2 Department of Anesthesiology, National Taiwan University Hospital, Taipei 100, Taiwan; 010388@ntuh.gov.tw (F.-F.T.); tslee@ntu.edu.tw (T.-S.L.); 3 Department of Surgery, National Taiwan University Hospital, Taipei 100, Taiwan; chienchiachen@ntuh.gov.tw

**Keywords:** lumboperitoneal shunt, hydrocephalus, nonintubated anesthesia

## Abstract

Lumboperitoneal shunt (LPS) implantation is a cerebrospinal fluid diversion therapy for the communicating type of normal-pressure hydrocephalus (NPH); NPH mainly affects older adults. However, endotracheal intubation for mechanical ventilation with muscle relaxant increases perioperative and postoperative risks for this population. Based on knowledge from nonintubated thoracoscopic surgery, which has been widely performed in recent years, we describe a novel application of nonintubated anesthesia for LPS implantation in five patients. Anesthesia without muscle relaxants, with a laryngeal mask in one patient and a high-flow nasal cannula in four patients, was used to maintain spontaneous breathing during the surgery. The mean anesthesia time was 103.8 min, and the mean operative duration was 55.8 min. All patients recovered from anesthesia uneventfully. In our experience, nonintubated LPS surgery appears to be a promising and safe surgical technique for appropriately selected patients with NPH.

## 1. Introduction

Normal-pressure hydrocephalus (NPH), presenting with cognitive decline, gait and balance impairments, and urinary incontinence, mainly affects the geriatric population. NPH is commonly treated with cerebrospinal fluid (CSF) shunting. The ventriculoperitoneal shunt (VPS) is currently the standard treatment, and the lumboperitoneal shunt (LPS), which diverts CSF from the spinal space in the lower back to the abdomen, is another choice for CSF diversion for the communicating type of hydrocephalus. Patients are routinely intubated for general anesthesia (GA) during these procedures. However, endotracheal intubation for mechanical ventilation with muscle relaxant increases the perioperative and postoperative risks for elderly persons [1,2,3], and this is a major concern during decision-making for this surgery. Therefore, the use of minimal sedation and the maintenance of spontaneous breathing throughout the operation have been applied in other surgeries such as video-assisted thoracoscopic surgery. Herein, we report a novel application of nonintubated LPS implantation in five patients with NPH.

## 2. Materials and Methods

The National Taiwan University Hospital Review Board approved this study. Between February 2019 and June 2019, five patients with a diagnosis of NPH underwent LP shunt placement by neurosurgeons. In all patients, a Strata^®^ (Medtronic, Inc., Goleta, CA, USA) Lumboperitoneal Adjustable Pressure valve set at 2.5 was implanted. Patients were sedated after anti-sialagogue (glycopyrrolate 0.2 mg) drugs routinely. All patients underwent preoperative and postoperative clinical evaluation by neurosurgeons in a single institution. Pre-operative lumbar CT was routinely performed to check for lumbar degenerative diseases that might hamper the insertion of LPS. Whole spine MRI was performed in cases when gait dysfunction due to cervical myelopathy needs to be ruled out. NPH was diagnosed if the patients presented with gait disturbance, urinary incontinence, or dementia, insidious onset and progression of symptoms over 3 months, CSF opening pressures between 70 and 245 mmH_2_O [4], and magnetic resonance imaging (MRI) or computed tomography (CT) demonstrated that ventricular enlargement was out of proportion when compared with the cerebral atrophy. Besides, CT should demonstrate an Evan’s index of at least 0.3. LPS implantation was performed in patients with a positive clinical response to a lumbar tap test. Other secondary causes of hydrocephalus were also ruled out. A whole-spinal-series CT or MRI was also performed to rule out spinal stenosis or other lesions that could block the patency of the CSF to LP shunt. Anesthesiologists were consulted for preoperative assessment of airway, and confirmed the feasibility of emergent endotracheal intubation in the lateral or supine positions if indicated.

### 2.1. Anesthesia

**Figure 1:**
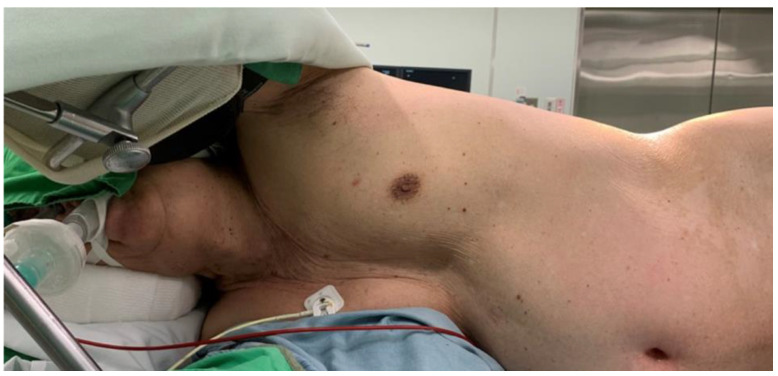
A patient was anesthetized with a laryngeal mask to maintain spontaneous breathing and was placed in a right decubitus position for lumboperitoneal shunt surgery.

**Figure 2:**
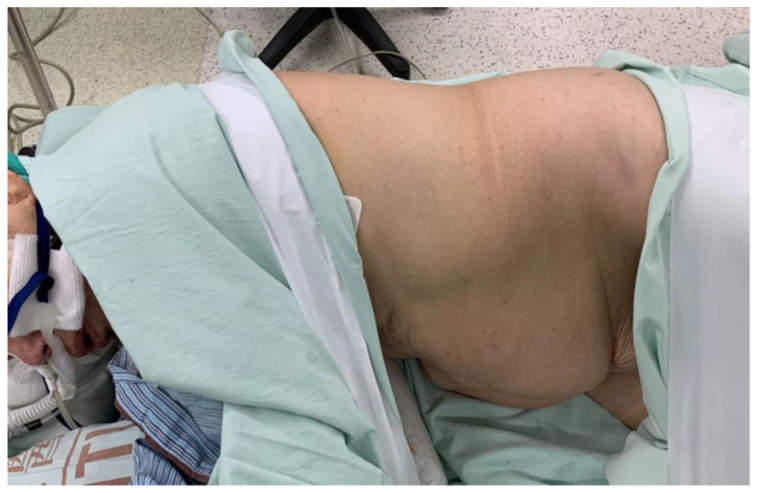
A patient was anesthetized with a high-flow nasal cannula to maintain spontaneous breathing and was placed in the right decubitus position for lumboperitoneal shunt surgery.

GA was performed with a laryngeal mask (LMA) in one patient and a high-flow nasal cannula (HFNC) in four patients to maintain spontaneous breathing during the surgery. The patients were started with standard hemodynamic monitoring including electrocardiography, blood pressure and respiratory assessment, and pulse oximetry. The bispectral index was also used to monitor the level of sedation. The target bispectral index value was between 50 and 60, and the probe was placed on the forehead of patients. End-tidal carbon dioxide was measured intranasally with a single-nostril catheter. We anesthetized the patients with propofol, starting with 25 μg/kg/min, and administered fentanyl at 2 μg/kg. After the patient was anesthetized, we applied an LMA (the size was decided by the body weight) or HFNC with 15 L/min of pure oxygen (Figure 1 and Figure 2).

During the procedure, sevoflurane was used for LMA, and propofol was used for HFNC. We carefully monitored the end-tidal carbon dioxide level to ensure the patient maintained spontaneous breathing. After the LP tube was inserted, weight-adjusted morphine was prescribed for postoperative pain control.

### 2.2. Operative Procedure

We used Medtronic Strata^®^ NSC LP adjustable pressure shunts for all patients, with the initial valve pressure setting at the highest pressure of 2.5, which is approximately 20 cmH_2_O. The patients were placed in the right decubitus position, and fluoroscopic guidance was used to localize the incision levels. Three skin incisions were made. A Tuohy needle was used to puncture the lumbar catheter insertion site, and a 2-cm skin incision was made caudal to the puncture site. A lumbar catheter was inserted into the subarachnoid space at the L3–S1 levels, mostly starting from the L4/5 level. The length of the lumbar catheter in the subarachnoid space was calculated preoperatively via CT or MRI to keep the catheter tip at about the L1/2 level. An anchor was used to hold the lumbar catheter, and sutures were used to fix the anchor to the fascia. A second skin incision was made over the left anterior flank, and a subcutaneous pocket was made to accommodate the pressure valve. At the same time, general surgeons made a third incision at the left upper quadrant of the abdomen to open the peritoneum for peritoneal catheter insertion. The lumbar catheter was tunneled from the lumbar incision to the flank incision, and the lumbar catheter was connected to the Strata^®^ NSC adjustable pressure valve. A subcutaneous tunneler was passed from the flank incision to the abdominal incision for the insertion of the peritoneal catheter. After confirming the patency of the abdominal catheter with respect to the flow of CSF by pressing on the valve, the 60-cm peritoneal catheter was placed inside the peritoneum. The valve was also sutured to the fascia with two stitches to prevent its migration.

The patients were moved to the postoperative recovery room after the surgery, and they were moved back to the neurosurgical ward for postoperative care.

### 2.3. Data Collection and Statistical Analyses

Baseline characteristics including age, sex, height, weight, diagnosis, underlying diseases, preoperative left ventricular ejection fraction, and methods of intraoperative oxygen delivery were extracted from chart review. The clinical data included intraoperative blood loss, anesthesia time, operative time, duration of hospital stay, and complications. Continuous data are presented as mean and standard deviation. Statistical analyses were conducted using SPSS 19.0 for Windows (IBM Corp., Armonk, NY, USA).

## 3. Results

Five patients, including three men and two women, with a mean age of 80 ± 10.8 years, underwent nonintubated LPS implantation between February and June 2019. The diagnosis was NPH in all patients via brain CT or MRI (Table 1). All patients underwent a preoperative evaluation of cardiac function by echocardiography. The average left ventricular ejection fraction was 62.5 ± 7.2%. The average anesthesia time and operative time were 103.8 ± 12.3 min and 55.8 ± 7.4 min, respectively. The average intraoperative blood loss was minimal (<50 mL). The average length of hospital stay was 2.8 ± 0.4 days. Oxygen delivery was achieved via LMA in one patient and HFNC in four patients. The oxygen saturation in these patients was maintained at levels above 90%, and there was no conversion to endotracheal intubation during the surgery. The extensive respiratory motion caused excessive abdominal wall motion, but the general surgeons overcame the difficulty. There was no in-hospital mortality, no surgical wound infection, and no shunt dysfunction at 1 year after the surgery. All patients experienced improvements in symptoms of ataxia.

## 4. Discussion

Our report describes five patients who underwent LP shunt implantation under GA without endotracheal intubation. To our knowledge, this is the first report of nonintubated LPS implantation. Our results of intraoperative PaO_2_ levels indicate that HFNC and LMA provided adequate oxygenation in BIS-targeted intravenous anesthesia. Considering the risk of GA in terms of age, the benefits of this GA type without endotracheal intubation are more prominent in certain diseases that mainly affect elderly people.

The non-intubated approach minimizes the adverse effects of tracheal intubation and GA, such as intubation-related airway trauma, ventilation-induced lung injury, residual neuromuscular blockade, and postoperative nausea and vomiting [5]. The recent trend of non-intubated thoracoscopic surgery has been safely and effectively used for wedge resection, lung volume reduction surgery, lung cancer surgery, bronchial sleeve resection, and even carina reconstruction, especially for high-risk intubated GA patients [5,6,7]. For old patients with severely compromised heart or lung function, or tracheal deformity, a non-intubated procedure could be considered as an alternative if LPS is indicated.

For the obstructive type of hydrocephalus, VPS is the preferred method of CSF diversion. However, nonintubated GA is not applicable to these patients because the procedure includes surgical draping that covers the head and face and tunneling of the catheter behind the ear with head rotation to the contralateral side, which makes airway protection difficult and conversion to endotracheal intubation impossible during surgery. For communicating hydrocephalus, both VPS and LPS are choices for shunt diversion. LPS has several advantages over VPS, including the avoidance of cranial surgery-related complications such as intracerebral hemorrhage or intraventricular hemorrhage due to ventricular puncture, as well as lower revision and infection rates [8,9]. To allow placement in the decubitus position throughout surgery, nonintubated GA is a feasible procedure in patients who undergo LPS implantation.

Traditional VP shunt needs subcutaneous tunneling through the head and neck area which made intubation unavoidable; however, general anesthesia with intubation can increase the risks of complications in patients with severely compromised heart or lung function (e.g., severe chronic obstructive pulmonary disease or congestive heart failure), graded as American Society of Anesthesiologists Classification (ASA) IV, or had high Charlson Comorbidity Index (CCI) score who easily suffer from aspiration pneumonitis or chocking after general anesthesia.

Complications of LPS implantation include shunt infection, shunt malfunction, and subdural hematoma or effusion due to overdrainage, as well as other complications due to abdominal procedures. Traditionally, the placement of the peritoneal catheter is completed by neurosurgeons through a mini-laparotomy incision. The potential complications of this procedure include visceral injury, subsequent abdominal hernia, wound infection, inadvertent subcutaneous placement, disconnection, and shunt obstruction [10,11,12]. Under nonintubated anesthesia, the difficulty of mini-laparotomy when patients have spontaneous breathing during the surgery should be overcome. A team approach involving an experienced general surgeon can facilitate the mini-laparotomy for the direct visualization of the catheter insertion and minimize complications, ensuring the proper function of the LP shunt system.

The length of the abdominal catheter, which affects resistance and change in the flow rate, can be shortened from the original length of 120 cm according to the neurosurgeons’ preference. Once overdrainage occurs in patients whose valve setting is at the highest pressure of 2.5, replacing the abdominal catheter with a longer one should be considered. In contrast, if underdrainage occurs when the valve setting is at the lowest pressure of 0.5, the abdominal catheter should be shortened by laparoscopic surgery.

Nonintubated anesthesia has been widely used in thoracic surgery in recent years with the benefits of enhancing postoperative recovery and reducing surgery-induced inflammatory responses [13,14,15]. Patients with severe respiratory disease have higher risks of ventilator dependency, perioperative and postoperative morbidity, and mortality after GA [1]. Compared to spontaneous respiration, positive pressure ventilation can significantly increase respiratory complications such as ventilation-induced lung injury [2,3]. From nonintubated video-assisted thoracoscopic surgery (VATS), it has been learned that nonintubated anesthesia, which maintains spontaneous respiration throughout surgery, can enhance postoperative recovery by preventing the complications of tracheal intubation and mechanical ventilation, and obviating the side effects of muscle relaxants [16]. Several reports in the literature have demonstrated that this technique can significantly shorten the length of hospital stay with a faster recovery when compared to intubated VATS [17,18,19].

Physiologically, the functional residual capacity falls below the closing capacity soon after anesthesia induction, which commonly leads to hypoxemia as a result of lung collapse and airway closure [20]. Nonintubated anesthesia could avoid excessively deep sedation, which may be associated with hypoxemia, hypoventilation, and hypotension. A difficult airway or potentially difficult intubation by preoperative assessment should be considered a relative contraindication to nonintubated anesthesia. If a stable surgical condition cannot be achieved or maintained throughout the surgery, anesthesiologists and neurosurgeons should evaluate the necessity and timing for conversion to conventional intubated shunt surgery. Secretions, saliva, or refluxed gastric contents can affect the vocal cords and patency of the airway, cause laryngospasms or bronchospasm, and enter the trachea and lungs with subsequent infection. Antisialagogue drugs may be used to reduce saliva production and decrease the risks of choking under nonintubated anesthesia. Despite the application of techniques for minimizing the degree of residual paralysis after GA such as pharmacological reversal and the use of intermediate-acting agents, about 33–64% of patients have evidence of inadequate neuromuscular recovery on arrival at the postoperative care unit [21,22]. By avoiding neuromuscular blocking agents, nonintubated anesthesia maintains spontaneous breathing during surgery and decreases the postoperative pulmonary complications associated with residual muscle relaxation, including postoperative hypoxemia and airway obstruction, especially in elderly patients receiving prolonged surgical procedures [23]. Opioids and sedatives were administered at a lower dose during the induction and maintenance of nonintubated anesthesia. Opioids are respiratory depressants that decrease the respiratory frequency and tidal volume [24]. Sedatives could cause hypoventilation due to upper airway obstruction and induce unconsciousness with a depressed respiratory drive [25]. Non-intubated anesthesia was reported to reduce inflammation based on postoperative white blood cell counts and tumor necrosis factor-α and C-reactive protein levels [26,27]. In summary, nonintubated surgery can minimize the residual effects of anesthetic agents, and patients can have adequate ventilation at the stage of postoperative care.

Nonintubated anesthesia provides tailored anesthetic combinations for select patients, which may require more experience, preparation, and vigilance compared to LPS implantation with intubated anesthesia. The feasibility of nonintubated anesthesia depends on the anesthesiologist’s comfort level, and the neurosurgeon’s and general surgeon’s experience. Considering the benefits and risks, patients with reduced heart or lung function could be good candidates for nonintubated LPS implantation [6]. NPH usually occurs in older adults, making the identification of comorbidities important before selecting the clinical management strategy. A preoperative lung function test and cardiac echocardiography can be arranged for pre-anesthetic assessment. Absolute contraindications for nonintubated anesthesia are the inexperience of the team members and patient refusal. Relative contraindications include obesity, previous abdominal surgery, and a relatively narrow interlaminar space of the lumbar spine, which could prolong the surgical time.

To keep the airway safe, operative duration is an important issue [28]. Performing a spinal tap for LPS implantation may be difficult in certain circumstances, including severe spinal stenosis, severe disc herniation, spinal tumor, interlaminar space stenosis, scoliosis, and congenital or acquired spinal deformity. In addition, marked stenosis of the cervical spinal canal can impede the flow of CSF from the brain to the LPS, which also makes the assessment of shunt patency during the surgery uncertain. Preoperative whole-spine MRI provides useful information on the anatomical status of CSF flow in the spinal canal, which could prevent prolonged surgery of the LPS implantation and increase the postoperative patency of the shunt. Lumbar spine CT provides information on the interlaminar space at different levels for the 14-gauge Tuohy needle insertion, and the interspinous space was also measured to decide whether the midline or paraspinal route would be used. Preoperative CT images help neurosurgeons to decrease the operative time by planning the best levels of insertion, and deciding the appropriate length of the lumbar catheter. The length can be estimated from the fascia layer to the center of the thecal sac at the level of insertion, and thereafter to the L1/2 level. According to a report, 15.8% of patients had their catheter tip at or above the L1/2 level, which could lead to injury to the conus medullaris [29]. A previous abdominal surgery or history of intra-abdominal adhesion should lead to prolonged surgery, and this should be evaluated by general surgeons in advance. These are all the critical points for preventing a prolonged operative time, which may increase the risks of nonintubated anesthesia.

Conversion to intubated GA in case of intraoperative emergency should be prepared in advance; and the coordination between surgeons, anesthesiologists, and nurses is key to success. The primary indications for conversion are persistent hypoxemia, tachypnea, a compromised airway, hemodynamic instability, and surgical issues such as challenging abdominal wall movement. As LPS surgery is performed in the right or left decubitus position, a large surgical drape should be prepared to cover the incised wounds over the lower back, flank, and abdominal wall to keep the wound sterile for a rapid change of positioning to perform intraoperative emergency intubation with the endotracheal tube. To execute emergent endotracheal intubation in the lateral position is another choice, which has been demonstrated to have high success rates and similar time required as supine position [30,31,32].

There are some limitations to our study. First, the number of patients was small because novel techniques were performed on selected patients to evaluate the feasibility and safety. Second, our study was retrospective. Randomized controlled trials are necessary to confirm the benefits of nonintubated LPS implantation.

## 5. Conclusions

Nonintubated LPS implantation demonstrated satisfactory postoperative outcomes. Nonintubated LPS surgery appears to be a promising and feasible surgical approach for appropriately selected patients with NPH.

## Figures and Tables

**Table 1:** The baseline characteristics and clinical data in these 5 NPH patients.

Case No.	Age/Sex	Diagnosis	Underlying Diseases	LVEF	Height, cm	Weight, kg	BMI, kg/m^2^	Oxygen Delivery	Anesthesia Time (Min)	Operative Time (Min)	Complications
1	78/M	NPH	Laryngeal cancer	62%	157.8	52.9	21.2	HFNC	98	48	None
2	87/M	NPH	Stroke, DM, Parkinson	51.8%	150	49	21.8	HFNC	123	59	None
3	66/M	NPH	MI, HTN, CKD	66.6%	168	51.9	18.4	HFNC	109	64	None
4	75/F	NPH	C-HIVD	71.2%	154	43	18.1	HFNC	94	60	None
5	94/F	NPH	Heart failure	61%	168	51.9	18.4	LMA	95	48	None

BMI: body mass index; C-HIVD: cervical herniated intervertebral disc; CKD: chronic kidney disease; DM: diabetes mellitus; HFNC: high-flow nasal cannula; HTN: hypertension; LMA: laryngeal mask; LVEF: left ventricular ejection fraction; MI: myocardial infarction; NPH: normal-pressure hydrocephalus.

## Data Availability

All relevant data are within the manuscript.
